# p53 attenuates acetaminophen-induced hepatotoxicity by regulating drug-metabolizing enzymes and transporter expression

**DOI:** 10.1038/s41419-018-0507-z

**Published:** 2018-05-10

**Authors:** Jiahong Sun, Yajie Wen, Yanying Zhou, Yiming Jiang, Yixin Chen, Huizheng Zhang, Lihuan Guan, Xinpeng Yao, Min Huang, Huichang Bi

**Affiliations:** School of Pharmaceutical Sciences, Sun Yat-Sen University, 510006 Guangzhou, China

## Abstract

Acetaminophen (APAP) overdose is the most frequent cause of drug-induced acute liver failure. Inhibition of APAP metabolic activation and promotion in APAP disposition are important to protect against APAP-induced liver injury. Tumor suppressor p53 is traditionally recognized as a surveillance molecule to preserve genome integrity. Recent studies have emerged on discovering its functions in metabolic regulation. Our previous study reported that p53 promoted bile acid disposition and alleviated cholestastic syndrome. Here, we examined the effect of doxorubicin (Dox)-mediated p53 activation on APAP-induced hepatotoxicity in mice and revealed a novel role of p53 in regulating APAP metabolism and disposition. Histopathological and biochemical assessments demonstrated that administration of Dox (10 mg/kg/d) before APAP treatment (400 mg/kg) significantly alleviated APAP-induced hepatotoxicity. Dox treatment prevented APAP-induced GSH depletion and lipid peroxidation. p53-null mice were more susceptible to APAP-induced liver injury. Further, we found that the expression of drug-metabolizing enzymes and transporters CYPs, SULTs and MRPs was regulated by p53. Dox treatment also promoted Nrf2 activation and increased the expression of Nrf2 target genes including GSTα/μ and NQO1, which contribute to APAP detoxification. Overall, this study is the first to demonstrate the protective role of p53 in regulating APAP metabolism and disposition, which provides a potential new therapeutic target for APAP-induced liver injury.

## Introduction

Acetaminophen (APAP) is a widely used analgesic and antipyretic drug, which is relatively safe and effective at therapeutic doses. However, APAP in high doses leads to hepatotoxicity, which is recognized as a major cause of acute liver failure^1^. Under normal condition, APAP is primarily catalyzed by UDP-glucuronosyltransferases (UGTs) and sulfotransferases (SULTs) to non-toxic metabolites^2^. An overdose of APAP saturates the glucuronidation and sulfation pathways, which is further converted to the reactive intermediate *N*-acetyl-*p*-benzoquinone imine (NAPQI) by cytochrome P450 (CYPs)^3^. NAPQI is normally detoxified by conjugation to glutathione (GSH), which is further excreted in urine by multidrug resistance-associated protein (MRP). However, the massive accumulation of NAPQI depletes GSH, which induces oxidative stress and ultimately lead to hepatocellular damage^4^. Glutathione S-transferases (GSTs) and NAD(P)H Quinone Dehydrogenase 1 (NQO1) are involved in the detoxification of APAP by regulating GSH homeostasis^5^. *N*-acetylcysteine has been clinically used as the primary antidote of APAP poisoning by replenishing GSH, which is only effective for the early stage of APAP intoxication^6^. Overall, the current therapeutic strategy for APAP-induced liver injury is not optimal. Inhibition of APAP metabolic activation and promotion in APAP disposition are an effective strategy to protect against APAP-induced liver injury.

Tumor suppressor protein p53 traditionally performs as a transcription factor in controlling genomic stability and cell growth^7^. p53 is activated upon a wide variety of stimuli, which include but are not limited to DNA damage. Our previous study also reported that p53 signaling pathway was associated with compensatory liver regeneration following APAP-induced liver injury^8^. Recently, accumulating findings revealed the regulatory role of p53 in various metabolic processes, such as glycolysis, oxidative phosphorylation, and lipid metabolism^9, 10^. Moreover, we recently revealed a novel role of p53 in regulating bile acid metabolism and disposition, which substantially prevented bile acid metabolic disorders^11^. On the basis of these data, we hypothesized that p53 regulates APAP metabolism and detoxification and further prevents APAP-induced hepatotoxicity. Thus, the current study aimed to explore whether p53 exerts the protective effect on APAP-induced hepatotoxicity and to determine the underlying mechanism involved in this hepatoprotection. The result demonstrated for the first time that p53 played a vital role in APAP-induced hepatotoxicity by regulating metabolizing enzymes and transporters related to APAP metabolism and detoxification.

## Results

### APAP-induced liver injury is attenuated by activation of p53 in mice

**Fig. 1:**
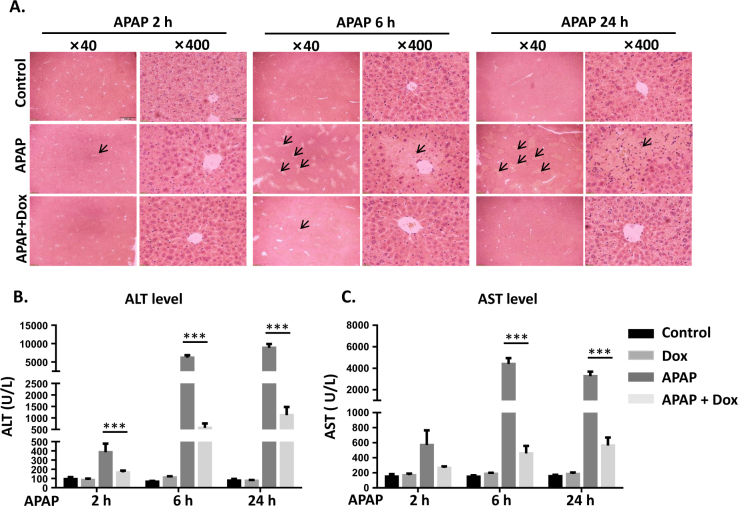
APAP-induced liver injury is attenuated by activation of p53 in mice. **a** H&E-stained liver sections were visualized at ×40 and ×400, *n* = 5. Activities of serum ALT (**b**) and AST (**c**) after Dox and APAP exposure. Data are presented as the mean ± SEM; *n* = 5–7. ^*^P < 0.05, ^**^P < 0.01, ^***^P < 0.001 versus APAP group

To investigate the effect of p53 activation on APAP-induced liver injury, mice were pretreated with Dox, a specific activator of p53, at 24 h prior to APAP administration. Histological analysis revealed that centrilobular necrosis was induced at 6 h after APAP treatment and peaked at 24 h. Much less hepatocellular injury and necrosis was observed in Dox/APAP group, indicating the protection of Dox against APAP-induced liver toxicity (Fig. 1a). Moreover, starting from 2 h after APAP treatment, a massive hepatic toxicity was induced as revealed by increased serum levels of ALT and AST. Co-treated with Dox significantly suppressed the elevation of ALT and AST levels. Compared to control, no significant change of ALT and AST activities was observed in Dox-treated group, indicating that the dosage of Dox used in this study did not induce liver toxicity (Fig. 1b, c).

### Activation of p53 by Dox prevents APAP-induced oxidative damage in liver

**Fig. 2:**
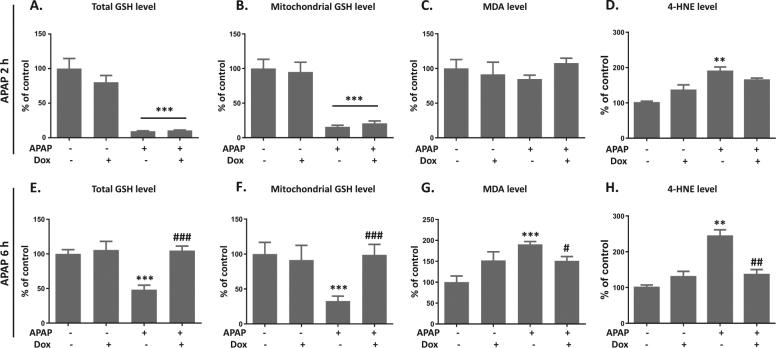
Activation of p53 by Dox prevents APAP-induced oxidative damage in liver. Total (**a** and **e**) or mitochondrial (**b** and **f**) GSH levels, MDA levels (**c** and **g**) and a4-HNE levels (**d** and **h**) at 2 or 6 h after APAP exposure were measured. Data are presented as the mean ± SEM; *n* = 5−6. ^*^P < 0.05, ^**^P < 0.01, ^***^P < 0.001

APAP-induced liver injury is triggered by GSH depletion, which further causes oxidative damage^12^. Thus, the effect of Dox on APAP-mediated oxidative stress in liver was explored. At 2 h after APAP exposure, both total and mitochondrial GSH levels were reduced to 10–20% of control, which was not preserved by Dox co-treatment (Fig. 2a, b). While GSH levels in the Dox/APAP group returned back to normal at 6 h after APAP exposure, Dox promotes the synthesis of GSH upon APAP challenge (Fig. 2e, f). Lipid peroxidation is a key index of APAP-mediated oxidative damage in liver^6^. Further, the effect of APAP and Dox on lipid peroxidation was determined by measuring MDA levels. No significant change was observed at 2 h after APAP treatment (Fig. 2c). A twofold increase of MDA level was induced by APAP at 6 h, which was attenuated by Dox (Fig. 2g). Similarly, APAP induced a significant upregulation of 4-HNE level, which was abolished by Dox treatment (Fig. 2d, h). Thus, we concluded that Dox prevented APAP-induced liver injury by promoting GSH recovery and suppressing lipid peroxidation.

### The severity of APAP-induced liver injury was enhanced in p53 knockout mice

**Fig. 3:**
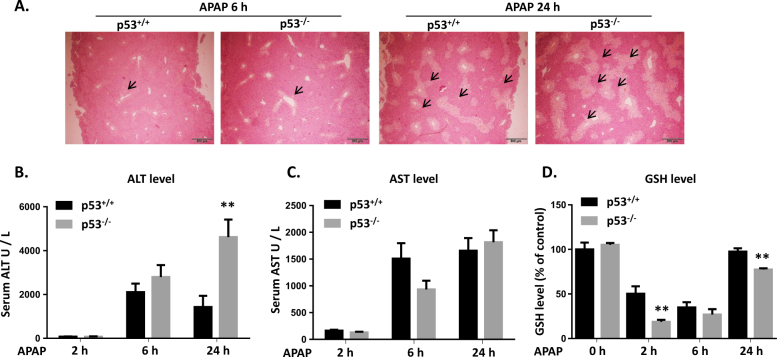
The severity of APAP-induced liver injury was enhanced in p53 knockout mice. **a** H&E-stained liver sections after 6 or 24 h after APAP exposure in p53^+/+^ and p53^−/−^ mice. Activities of serum ALT (**b**), AST (**c**) and GSH (**d**) after APAP exposure. Data are presented as the mean ± SEM; *n* = 5–6. ^**^P < 0.01 versus p53^+/+^ group

To further explore the role of p53 in APAP-induced liver injury, p53^−/−^ and p53^+/+^ mice were treated with APAP and the severity of liver injury was compared. Histological analysis demonstrated that massive hepatic toxicity was observed in p53^−/−^ mice at 6 h after APAP exposure. Compared to p53^+/+^ mice, a more serious liver injury was induced by a 24-h exposure of APAP in p53^−/−^ mice (Fig. 3a). Although no significant difference of ALT level was observed between p53^−/−^ and p53^+/+^ mice at 2 and 6 h after APAP treatment, ALT level in p53^−/−^ group was threefold higher than that in p53^+/+^ group by 24 h (Fig. 3b). For AST level, no significant difference was observed in these two groups (Fig. 3c). GSH level in p53^−/−^ mice was significantly lower than that in p53^+/+^ mice at 2 and 24 h after APAP exposure, indicating a more serious oxidative stress induced by p53 deficiency (Fig. 3d). Overall, these results indicated that p53-null mice were more susceptible to APAP-induced liver injury.

### p53 regulates gene expression of drug-metabolizing enzymes and transporters

**Fig. 4:**
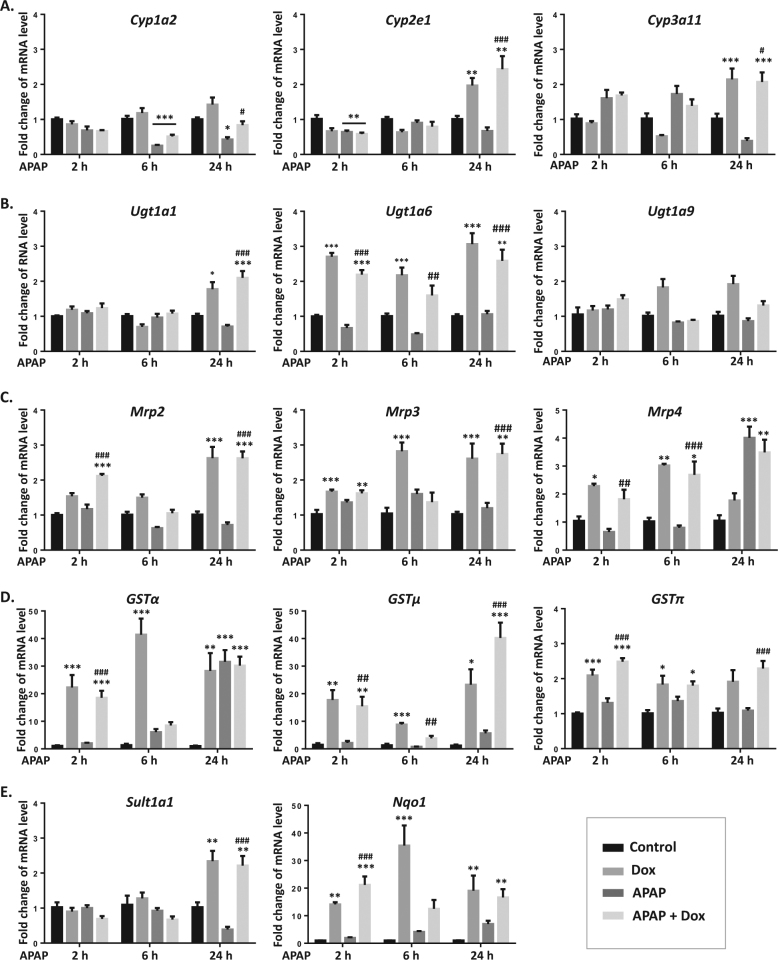
Dox regulates gene expression of drug-metabolizing enzymes and transporters. **a**−**e** mRNA expression of *Cyp1a1/2e1/3a11, Ugt1a1/6/9, Mrp2/3/4, Gstα/μ/π, Sult1a1, and Nqo1* were measured after Dox and APAP treatment. Data are the mean ± SEM; *n* = 5. ^*^P < 0.05, ^**^P < 0.01, ^***^P < 0.001 versus control group; ^#^*P* < 0.05, ^##^*P* < 0.01, ^###^*P* < 0.001 versus APAP group

A number of metabolizing enzymes and transporters participated in APAP metabolism and elimination, which were time-dependently measured after Dox and APAP treatment. CYP isoforms such as CYP2E1, CYP3A4 (human ortholog of CYP3A11) and CYP1A2 play the most important role in APAP bioactivation. Interestingly, we found that gene levels of *Cyp1a1/2e1/3a11* were reduced by APAP and upregulated by Dox treatment (Fig. 4a). The gene expression of *Ugt1a1/6 and Sult1a1* was elevated by Dox and was not regulated by APAP (Fig. 4b, e). As shown in Fig. 4c, the expression of *Mrp2/3/4* were also significantly upregulated in both Dox group and Dox/APAP group. A 24-h treatment of APAP also increased *Mrp4* mRNA level. There was a more than 20-fold increase of *Gstα/μ* and *Nqo1* mRNA levels in Dox and Dox/APAP groups. Notably, a mild upregulation of *Gstα/μ* and *Nqo1* expression was also induced by APAP at 24 h (Fig. 4d).

**Fig. 5:**
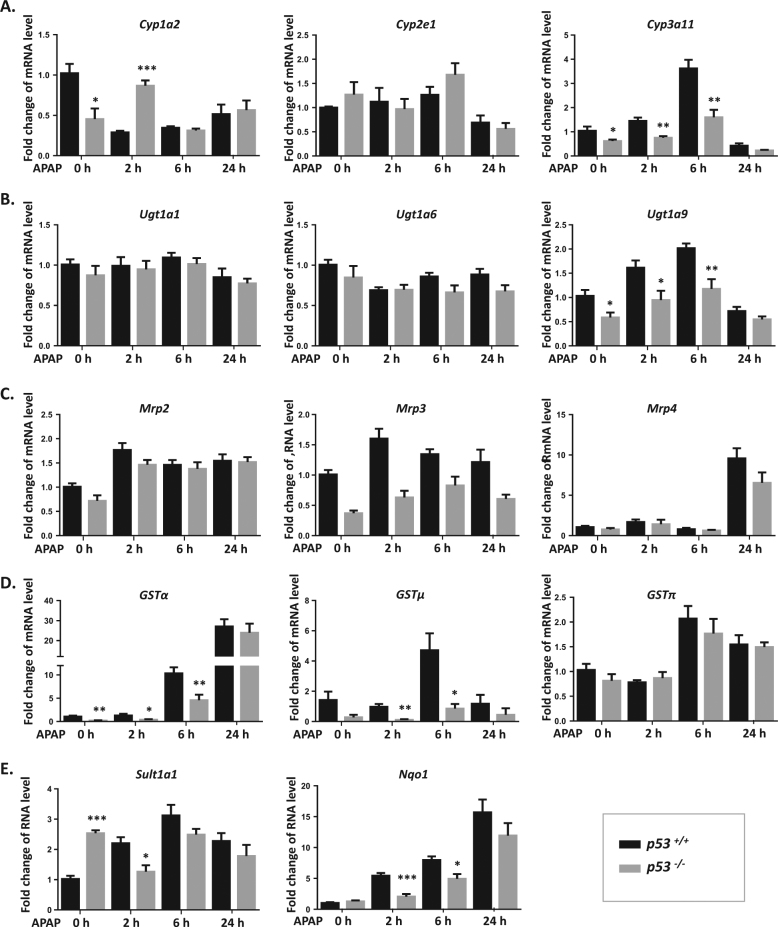
Gene expression of drug-metabolizing enzymes and transporters after APAP exposure in p53^+/+^ and p53^−/−^ mice. **a**−**e** mRNA expression of *Cyp1a1/2e1/3a11, Ugt1a1/6/9, Mrp2/3/4, Gstα/μ/π, Sult1a1*, *and Nqo1* were measured after APAP treatment in p53^−/−^ and p53^+/+^ mice. Data are the mean ± SEM; *n* = 5. ^*^P < 0.05, ^**^P < 0.01, ^***^P < 0.001 versus p53^+/+^ group

To further confirm the regulation of p53 on APAP metabolism and elimination, we compared the gene expression of these metabolizing enzymes and transporters between p53^−/−^ and p53^+/+^ mice. Compared to p53^+/+^ mice, mRNA levels of metabolizing enzymes *Cyp1a2/3a11* and *Ugt1a9* were lower in p53^−/−^ mice. No significant difference of *Cyp2e1* and *Ugt1a1/6* expression was observed between p53^−/−^ and p53^+/+^ mice (Fig. 5a, b). The mRNA level of *Sult1a1* in p53^−/−^ mice was higher than that in p53^+/+^ mice, which was reduced by APAP at 2 h (Fig. 5e). There was no change of *Mrp2/3/4* expression in p53^−/−^ mice (Fig. 5c). The expression of *Gstα/μ and Nqo1* was significantly decreased in p53^−/−^ mice, which was opposite with the change after Dox treatment. Interestingly, an upregulation of *Mrp4*, *GSTα,* and *Nqo1* levels was induced by APAP regardless of p53 status, indicating that APAP itself also regulates expression of these drug-metabolizing enzymes and transporters (Fig. 5c−e).

### p53 regulates protein expression of enzymes related to APAP metabolism and detoxification

**Fig. 6:**
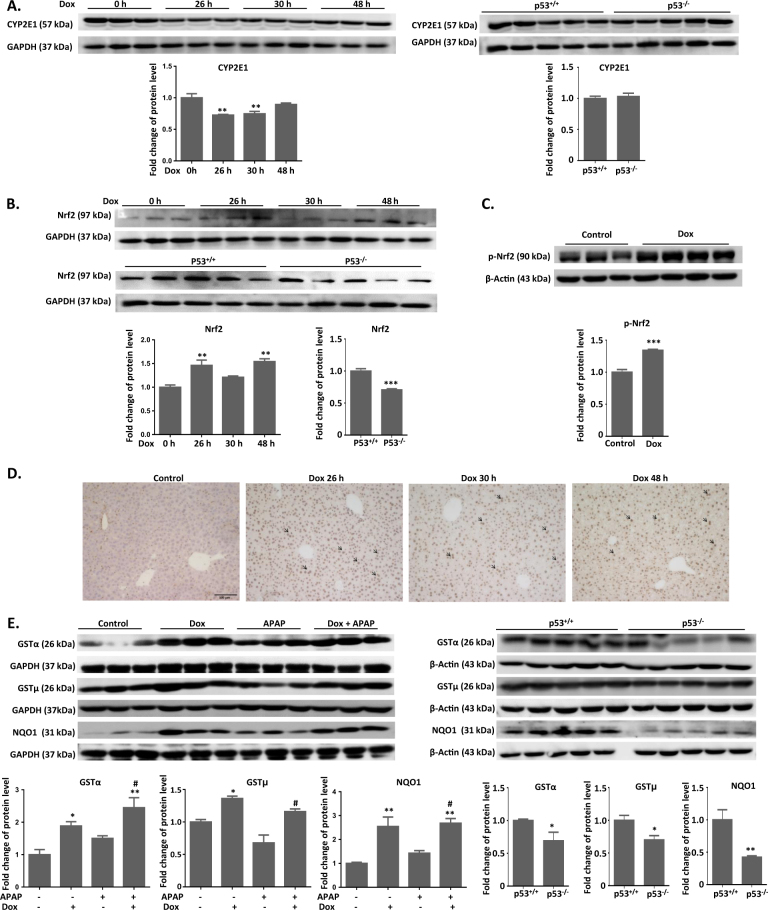
Protein expression of CYP2E1, Nrf2, GSTα/μ, and NQO1 after APAP/Dox exposure in p53^+/+^ and p53^−/−^ mice. **a**, **b** Liver samples were subjected to immunoblot with antibodies specific for CYP2E1 and Nrf2 after Dox treatment alone and in p53^−/−^ or p53^+/+^ mice. **c** Liver samples were subjected to immunoblot with antibodies specific for phosphorylation of Nrf2 after Dox exposure. **d** Immunohistochemical staining of Nrf2 in liver section after Dox exposure. **e** Liver samples were subjected to immunoblot with antibodies specific for GSTα/μ and NQO1 after Dox and APAP exposure and in p53^−/−^ or p53^+/+^ mice. Quantitation of CYP2E1, GSTα/μ, and NQO1 was normalized to GAPDH or β-actin. Bars represent normalized relative densities plotted as mean ± SEM, ^*^P < 0.05, ^**^P < 0.01 versus control or p53^+/+^ group; ^#^*P* < 0.05 versus APAP group

The metabolic activation of APAP is initiated by CYP-mediated conversion to NAPQI, which is the most proximal event in the toxicity mechanism. CYP2E1 plays the most important role in APAP bioactivation. Here we measured the protein expression of CYP2E1 after p53 activation and depletion. Since mice with a 24-h pre-treatment of Dox were collected at 2, 6, and 24 h after APAP exposure, we parallelly measured the expression of Nrf2 at 26, 30, and 48 h after Dox treatment alone. As shown in Fig. 6a, Dox treatment alone induced a significant downregulation of CYP2E1 level at 26 and 30 h. No significant difference of CYP2E1 expression was observed between p53^+/+^ and p53^−/−^ mice (Fig. 6a).

Among all the drug-metabolizing enzymes and transporters regulated by p53, the change of *Gstα/μ* and *Nqo1* mRNA expression was the most significant, which are well-known Nrf2 downstream target genes^5, 13^. We first investigated the regulation of p53 on Nrf2 signaling pathway. Nrf2 mRNA and protein levels were elevated by Dox treatment, which were reduced by p53 depletion (Fig. 6b and Supplementary Fig. 1). Also, a 24-h exposure of Dox promoted the phosphorylation of Nrf2 (Fig. 6c). As shown in Fig. 6d, Dox alone induced the translocation of Nrf2 into nucleus, which further confirmed the activation of Nrf2 signaling pathway. We further measured the protein expression of GSTα/μ and NQO1 after p53 activation or inhibition. At 24 h after APAP exposure, GSTα/μ and NQO1 protein levels were significantly elevated in Dox and Dox/APAP groups (Fig. 6e). The protein expression of GSTα/μ and NQO1 in p53^−/−^ mice was lower than that in p53^+/+^ mice (Fig. 6e). However, a slight upregulation of GSTα/μ and NQO1 protein expression was also observed in APAP group, which was consistent with the change of mRNA levels (Fig. 6e).

### Activation of p53 prevents APAP-induced toxicity in vitro by promoting Nrf2 activation and its downstream target gene expression

**Fig. 7:**
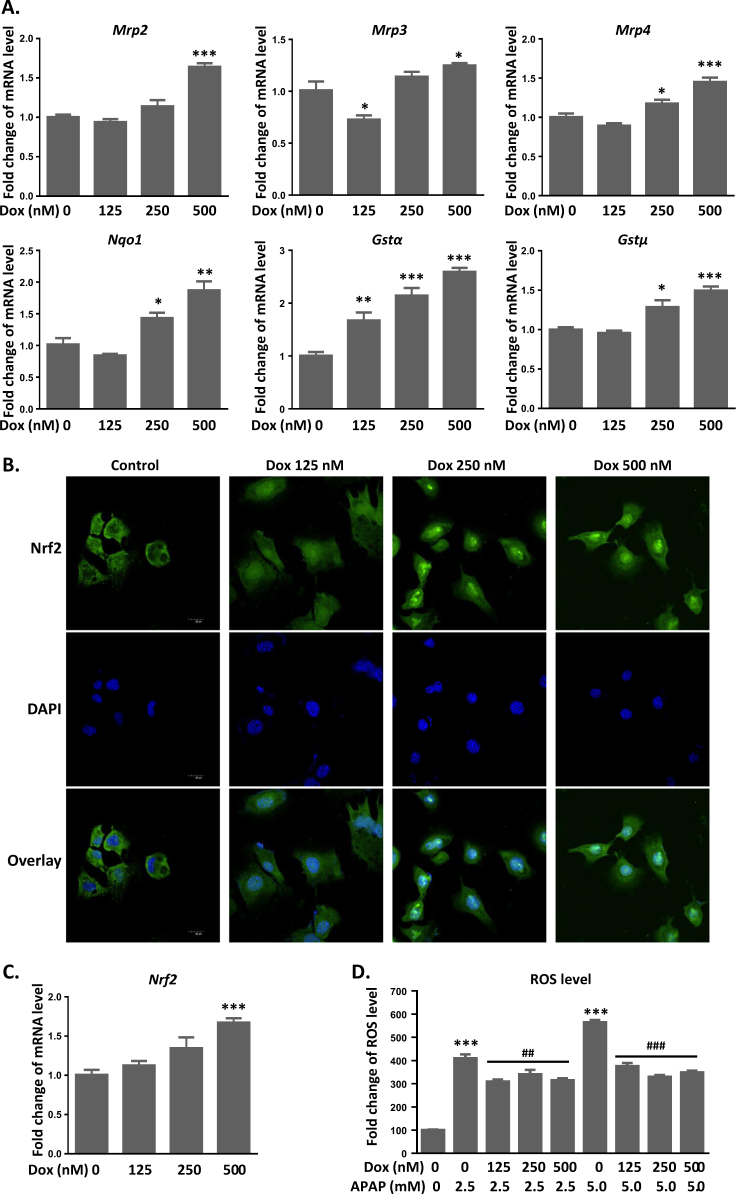
Activation of p53 prevents APAP-induced toxicity in vitro by promoting Nrf2 activation and its downstream target gene expression. a mRNA expression of Mrp2/3/4, *Gstα/μ*, and *Nqo1* was measured after Dox treatment in AML12 cells. **b** Immunocytochemistry for Nrf2 (Green) and DAPI (blue). Images were captured at 24 h of exposure to Dox by confocal microscopy. **c** mRNA expression of *Nrf2* was measured after a 24-h Dox treatment. **d** ROS levels were detected by H_2_DCF, and fluorescence was measured by plate reader at 48 h after APAP and Dox exposure. Data are the mean ± SEM; *n* = 5. ^*^P < 0.05, ^**^P < 0.01, ^***^P < 0.001 versus control group; ^##^*P* < 0.01, ^###^*P* < 0.001 versus APAP group

To further elucidate the protective mechanism of p53 against APAP toxicity, we treated AML12 cells with Dox (125–500 nM) and also found a dose-dependent upregulation of mRNA levels of *Mrp2/3/4, Nqo1*, and *GSTα/μ* (Fig. 7a). Next, we investigated if the protective effect of p53 worked through the activation of Nrf2. There was a clear translocation of Nrf2 (Fig. 7b, green) into the nucleus (Fig. 7b, blue) at 24 h after Dox exposure, which is necessary for Nrf2 activation. Moreover, the mRNA expression of *Nrf2* was elevated by Dox treatment, indicating that p53 enhanced the activity of Nrf2 signaling pathway (Fig. 7c). Further, AML12 cells was exposed with APAP (2.5–5 mM) and Dox, and determined whether Dox inhibits APAP-induced intracellular ROS accumulation. Similarly, intracellular ROS level was elevated by fourfold after a 48-h exposure of APAP, which was ameliorated by the simultaneous treatment of Dox (Fig. 7d).

**Fig. 8:**
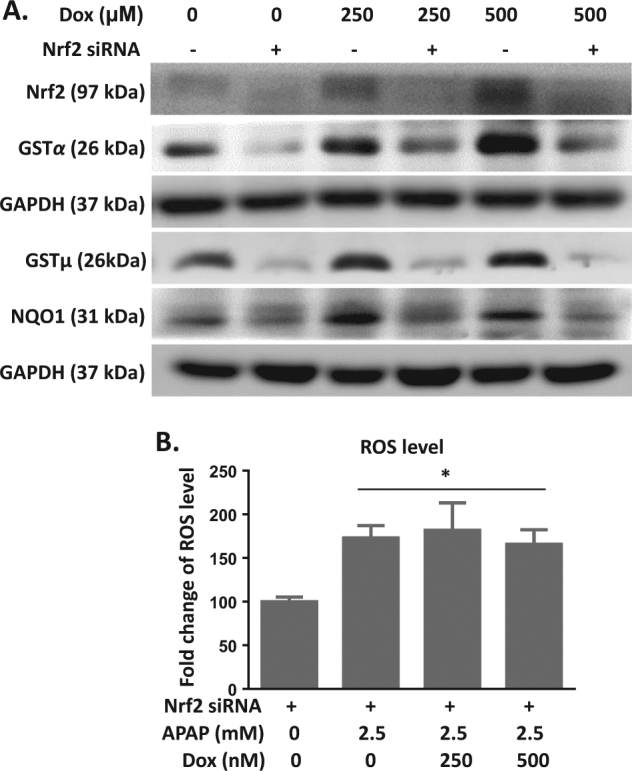
The protective effect of p53 against APAP toxicity is Nrf2-dependent. **a** AML12 cells were transfected with scrambled and Nrf2 siRNA for 36 h and further treated with Dox for 48 h. Cell samples were subjected to immunoblot with antibodies specific for GSTα/μ and NQO1. **b** AML12 cells were transfected with Nrf2 siRNA for 48 h and further treated with APAP and Dox for 24 h. Intracellular ROS were measured by H_2_DCF. Data are the mean ± SEM; *n* = 5. *P < 0.05 versus control group

To characterize the role of Nrf2 in Dox-mediated protection against APAP toxicity, AML12 cells were transfected with siRNA targeting Nrf2. Nrf2 mRNA expression was reduced by 50% at 48 h and by 70% at 72 h after transfection (Supplementary Fig. 2). Moreover, Nrf2 siRNA transfection significantly reduced NRF2 protein expression. Dox treatment induced an upregulation of NRF2 protein level in scrambled siRNA-transfected cells. Dox increased protein levels of NQO1 and GSTα/μ in scrambled siRNA-transfected cells, which was abolished by Nrf2 knockdown (Fig. 8a). Further, we investigated the protection efficacy of Dox in Nrf2-knockdown AML12 cells. After 48 h of Nrf2 siRNA transfection, cells were treated with APAP and Dox for 24 h and intracellular ROS levels were measured. Compared to normal AML12 cells, the inhibition of APAP-induced ROS accumulation by Dox was attenuated by Nrf2 silencing (Fig. 8b).

## Discussion

The present study demonstrated the protective effect of p53 on APAP-induced liver toxicity by regulating the expression of drug-metabolizing enzymes and transporters, which enhanced APAP metabolism and suppressed oxidative damage. p53-null mice were more susceptible to APAP-induced liver injury. Moreover, p53 promoted Nrf2 activation and the transcription of its downstream target genes related to APAP detoxification.

Under the catalysis of UGTs and SULTs, APAP is primarily converted to pharmacologically inactive glucuronide and sulfate conjugates, which is further excreted into the urine or bile by MRPs^2^. An increase of UGTs, SULTs, and MRPs expression was observed after p53 activation, indicating that Dox promotes the conversion of APAP to non-toxic metabolites as well as the elimination of APAP metabolites. The metabolic activation of APAP is initiated by CYP-mediated conversion to NAPQI, which is the most proximal event in the toxicity mechanism^14^. NAPQI causes oxidative stress and binds covalently to liver proteins, which is normally detoxified by GSH both nonenzymatically and enzymatically in a reaction catalyzed by GSTs^15, 16^. In this study, we found that a slight increase of these CYPs mRNA expression was induced by Dox. p53 deficiency did not induce a significant change of CYP2E1 protein expression, which was consistent with previously published study^17^. While Dox treatment suppressed the CYP2E1 level, activation of p53 might inhibit APAP bioactivation. There was also a profound increase of GSTs expression after Dox exposure. Moreover, NQO1, which controls redox homeostasis and facilitates adaptation of cells to oxidative stress, was also upregulated by p53 activation. The higher levels of these enzymes that are responsible for APAP detoxification account for the more effective protection by intracellular antioxidants to APAP challenge. Therefore, we concluded that activation of p53 effectively prevents APAP accumulation by promoting APAP metabolism and elimination. It is important to note that the expression of several enzymes was upregulated by Dox, which was not significantly changed in p53^−/−^ mice. This observation was consistent with our previously published results^11^. We found that Dox induced the upregulation of MRP2/3/4 protein expression, which was abolished by p53 deficiency, indicating the p53-dependent regulation of these enzyme expressions by Dox. Only MRP4 expression was reduced by p53 deficiency. There was no significant difference of MRP2/3 levels between p53^+/+^ and p53^−/−^ mice. We speculated that many signaling pathways (Nrf2, PI3K/Akt, and MEK/ERK signaling pathways) also have been shown to involve in the regulation of MRPs expression, which induced a compensatory regulation of these enzymes in response to the depletion of p53^18, 19^.

An elevation of ALT level was induced at 2 h after APAP exposure, which was prevented by Dox. GSH depletion induced by APAP was not reversed by Dox. Also, no significant change of lipid peroxidation was observed after APAP and Dox treatment. In contrast, both ALT and AST levels were dramatically reduced by Dox at 6 h after APAP exposure. Moreover, Dox prevented APAP-induced GSH depletion and accumulation of lipid peroxidation, indicating that the maximum protective effect of Dox was achieved at 6 h after APAP exposure. Starting from 2 h after APAP exposure, a more than tenfold increase of GSTs and NQO1 levels was induced by Dox, which led us to investigate whether these detoxifying enzymes play a major role in the defense against APAP toxicity.

Nrf2 regulates the expression of a battery of cytoprotective genes encoding intracellular detoxifying enzymes, including MRPs, NQO1, and GSTs, which are responsible for APAP elimination and detoxification^20^. It has been known that activation of Nrf2 confers potent resistance against acute drug toxicity^5^. Our results showed that Dox increased Nrf2 expression and promoted the activation and translocation of Nrf2 into the nucleus, indicating that p53 enhanced the activity of Nrf2 signaling pathway. Previous published study also reported that p21, a p53 target gene stabilizes Nrf2 by binding to Keap1 and interfering with its ability to promote Nrf2 ubiquitylation and proteasomal degradation^21^. In light of the role of Nrf2 in the protection of p53 against APAP-induced liver damage, Nrf2 was depleted using siRNA transfection to study whether lack of this transcription factor renders hepatocytes more vulnerable to APAP toxicity after p53 activation. Our results illustrated that Dox suppressed APAP-induced ROS accumulation, which was abolished by Nrf2 silencing. Moreover, Dox failed to upregulate the expression of NQO1 and GSTs in Nrf2-knockdown hepatocytes. Overall, our study allows us to speculate that the protective effect of Dox is achieved by promoting Nrf2 activation and the transcription of its downstream target genes related to APAP detoxification. It is very important to note that the Nrf2 signaling pathway may not be the only factor contributing to the protection of Dox against APAP-induced liver injury. Our previous study demonstrated that p53 directly regulates metabolizing enzymes and transporters such as CYP2B6 and MRP3, which are related to APAP metabolism and detoxification^11^. Published study also reported that p53 directly activated CYP3A4 transcription^22^. In the current study, CYPs related to APAP bioactivation was also found to be regulated by p53. Therefore, p53 might also directly regulate the transcription of CYPs related to APAP metabolism, which needs to be addressed in future studies.

p53 is a tumor suppressor that plays an important role in regulating cell growth, DNA repair, and apoptosis. Under severe DNA damage by APAP overdose, p53 is activated to inhibit cell proliferation or trigger cell apoptosis^22^. Moreover, our previous study also reported that dynamic and coordinated regulation of Nrf2 and p53 signaling pathways was associated with compensatory liver regeneration after APAP-induced acute liver injury^8^. As shown in Supplementary Fig. 3, the expression of core cell cycle protein CDK4 and cyclin D1 was lower in p53^−/−^ mice than that in p53^+/+^ mice. Therefore, suppression of p53 activity might prevent cell apoptosis and promote compensatory liver regeneration, which might contribute to a relatively mild liver injury at early time points of APAP exposure^23^. Consistent with published study, APAP induced a more severe damage in liver at 24 h after APAP exposure^20^. We speculated that suppression of p53 fails to enhance APAP metabolism and detoxication regulated by metabolizing enzymes and transporters, which was positively correlated with this exacerbated liver damage.

Overall, this experiment was done in an effort to comprehensively investigate the regulatory role of p53 on APAP metabolism. This study is the first to demonstrate that p53 prevents against APAP-induced liver injury by regulating the metabolizing enzymes and transporters related to APAP detoxification and elimination. These significant data may provide a clinically relevant argument for using p53 against APAP-induced acute liver injury.

## Methods

### Animal experiments

Male C57BL/6 mice (6–8 weeks old) were obtained from Laboratory Animal Center of Sun Yat-Sen University (Guangzhou, China). p53 knockout and paired wild-type mice (19–20 g) were established by NIFDC and Beijing Biocytogen Co., Ltd and purchased from the National Center of Laboratory Rodents. All animals were maintained under controlled conditions (22–24 °C, 55–60% humidity, and 12-h light/dark cycle) with free access to standard food and water. All procedures followed the Regulations of Experimental Animal Administration issued by the Ministry of Science and Technology of the People’s Republic of China. Animal protocols were approved by the Ethics Committee on the Care and Use of Laboratory Animals of Sun Yat-Sen University. All animals were fasted 3 h or overnight before APAP administration, since the severity of APAP-induced could be regulated by fasting period^24^. A relative mild liver injury model was used in p53^+/+^ and p53^−/−^ mice by reducing the fasting time to 3 h. Dox (Aladdin Company, Shanghai, China) at 10 mg/kg or saline was administered to WT mice by intraperitoneal injections at 24 h before APAP administration. APAP (Sigma-Aldrich, St. Louis, MO) solution was made fresh in 0.9% saline at 40 mg/ml, and mice were administered a single dose of 400 mg/kg APAP by intraperitoneal injection. Mice were killed at 0, 2, 6, and 24 h after APAP treatment. Serum samples and liver tissues were harvested. Tissues were flash frozen in liquid nitrogen and stored at –80 °C for further use.

### Cell culture and treatment

AML-12 cells, a nontumorigenic mouse hepatocyte cell line, were obtained from the American Type Culture Collection (ATCC, Manassas, VA) and cultured in DMEM/F12 containing 0.005 mg/ml insulin, 0.005 mg/ml transferrin, 5 ng/ml selenium, 40 ng/ml dexamethasone and 10% FBS at standard cell culture conditions (5% CO_2_, 95% air). 100 nM negative small interference RNA (siRNA) or siRNA targeting at Nrf2 (RiboBio, Guangzhou, China) were transiently transfected using lipofectamine 2000 siRNA Transfection Reagent (Roche diagnostics, Mannheim, Germany). Cells were treated with Dox (125−500 nM) or APAP (2.5−5 mM) at 48 h after siRNA transfection and incubated for 24−48 h.

### Histologic evaluation

Tissues were immediately fixed in formaldehyde, embedded in paraffin, sectioned, and stained with hematoxylin and eosin stain (H&E) according to a standard protocol. H&E-stained liver sections were examined using a Leica DM5000B microscope (Leica, Heidelberg, Germany).

### Biochemical evaluation

APAP-induced liver injury was evaluated by measuring serum alanine transaminase (ALT) and aspartate transaminase (AST) catalytic activities. ALT and AST levels were analyzed using commercial kits (Kefang Biotech, Guangzhou, China) on a Beckman Synchron CX5 Clinical System (Beckman Coulter, Brea, CA) according to the manufacturer’s protocol. To assess lipid peroxidation induced by APAP, levels of malondialdehyde (MDA) were also determined using commercially available kits (Nanjing Jiancheng Bioengineering Institute, Nanjing, China).

### GSH measurement

GSH levels of total or mitochondria extracts in the liver were measured using a GSH assay kit (Nanjing Jiancheng Bioengineering Institute, Nanjing, China). Liver mitochondria were isolated by differential centrifugation following the assay kit’s instruction (Sangon Tech).

### qRT-PCR analysis

qRT-PCR analysis for the expression of target genes in mouse livers and AML12 hepatocytes was performed as described previously^25^. The sequences of gene-specific primers were listed in Supplementary Table 1.

### Western blot analysis

Proteins extracted from mice liver tissue or AML12 hepatocytes were prepared using radioimmunoprecipitation assay lysis buffer (Biocolor BioScience and Technology, Shanghai, China) and were determined by the BCA Protein Assay (Thermo Scientific, Rockford, IL). Protein extract was separated in 8–12% SDS-PAGE and electrophoretically transferred onto polyvinylidene fluoride membranes. After blocking with 5% non-fat dry milk in Tris-buffered saline, membranes were incubated overnight with primary antibodies, including NRF2 (Cell Signaling Technology, Shanghai), phosphate NRF2 (Abcam), CYP2E1 (Cell Signaling Technology, Shanghai), NQO1(Sangon Biotech, Shanghai), GSTα (Sangon Biotech, Shanghai), GSTμ (Sangon Biotech, Shanghai), CDK4 (Sangon Biotech, Shanghai), Cyclin D1 (Cell Signaling Technology, Shanghai), β-actin (Santa Cruz Biotechnology, Dallas), and GAPDH (Santa Cruz Biotechnology, Dallas, TX). A secondary horseradish peroxidase-conjugated anti-rabbit or anti-mouse IgG antibody (Santa Cruz Biotechnology, Dallas) was subsequently applied, and then specific bands were visualized using an enhanced chemiluminescence detection kit (Engreen Biosystem, Beijing, China).

### Immunocytochemistry

AML-12 cells were fixed with 4% paraformaldehyde (Sigma-Aldrich) in PBS for 15 min after respective exposures. The cells were permeabilized with 0.25% Triton X-100 for 10 min and were then incubated in 10% serum (Sigma-Aldrich) blocking solution containing 0.3 M glycine for 30 min. Cells were exposed to anti-Nrf2 antibody (1:100 in blocking solution; Santa Cruz Biotechnology, Dallas) overnight at 4 °C, followed by incubation with appropriate fluorescence-conjugated secondary antibodies (Molecular Probes) for 1 h. Nuclei were counterstained with 49,6-diamidino-2-phenylindole (DAPI). Images were acquired using a fluorescence confocal microscope (Zeiss Violet Confocal; Zeiss, Oberkochen, Germany) with a ×40 objective.

### ROS detection

Changes in intracellular ROS were measured by the ROS-reactive fluorescent indicator 29,79-dichlorodihydrofluorescein diacetate (H_2_DCFDA) (Molecular Probes). After respective exposures, the medium was removed and the cells were washed once with PBS and then incubated with 10 mM H_2_DCFDA for 30 min at 37 °C. Mean fluorescence intensity of DCF was measured using the BioTek Synergy H1 Hybrid plate reader (excitation, 485 nm; emission, 530 nm). DCF fluorescence was standardized based on cell viability.

### Statistical analysis

The data are shown as means ± SEM. Statistical analyses were performed using one-way analysis of variance with Tukey’s post-hoc test. GraphPad Prism 5.0 (GraphPad Software, Inc., La Jolla, CA) was used for statistical analyses.

## Electronic supplementary material

Sup Fig 1

Sup Fig 2

Sup Fig 3

Sup Fig legend

Sup Table 1
