# Supplementary figures and images for: p53 attenuates acetaminophen-induced hepatotoxicity by regulating drug-metabolizing enzymes and transporter expression
Source: Cell Death Dis. 2018 May 10;9(5):536. doi: 10.1038/s41419-018-0507-z (PMC5945795; doi:10.1038/s41419-018-0507-z)

A.

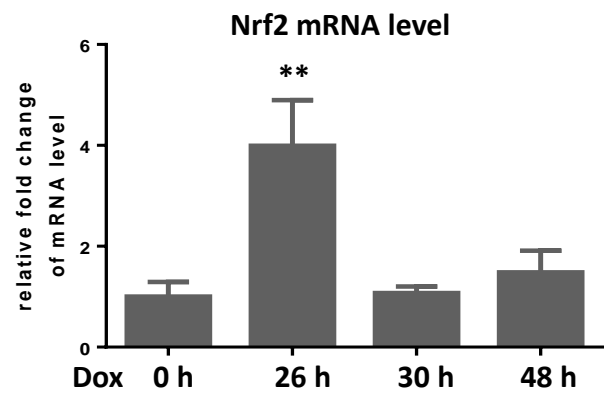

B.

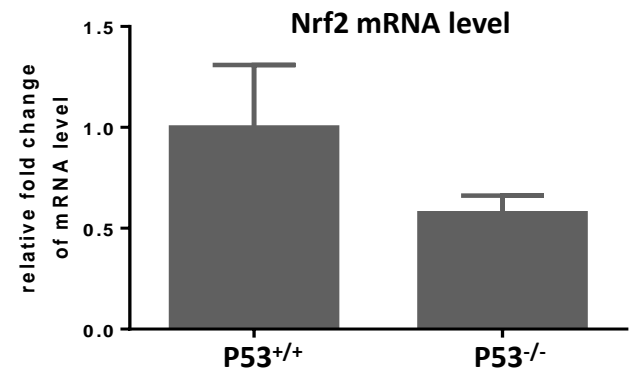

Supplement: Supplementary file 1 — Sup Fig 1 [file 41419_2018_507_MOESM1_ESM.pdf]

Sup Fig 2

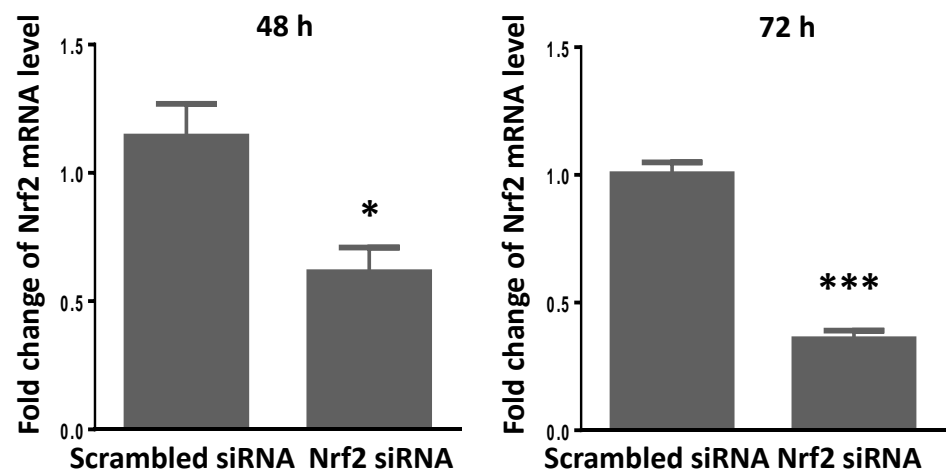

Supplement: Supplementary file 2 — Sup Fig 2 [file 41419_2018_507_MOESM2_ESM.pdf]

Sup Fig 3

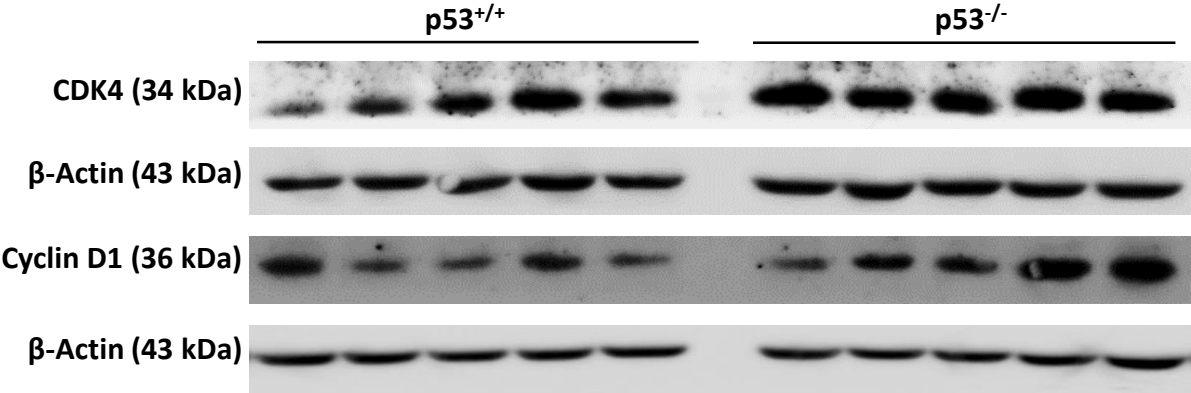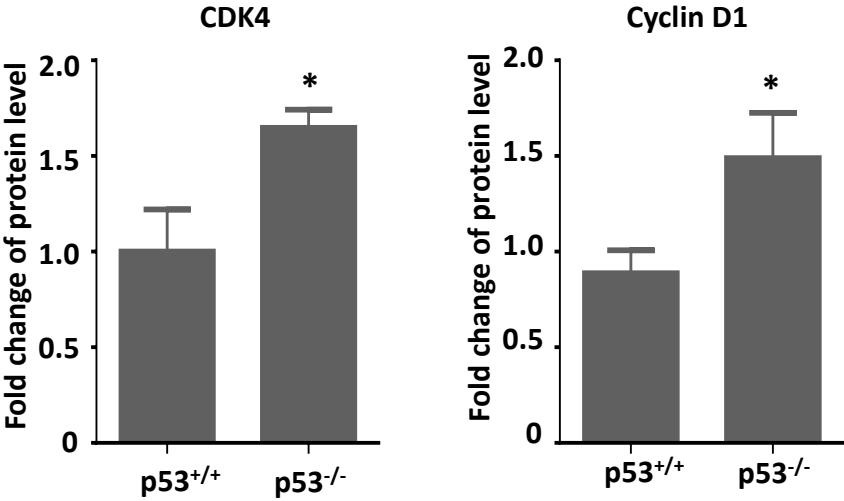

Supplement: Supplementary file 3 — Sup Fig 3 [file 41419_2018_507_MOESM3_ESM.pdf]
