# Supplementary material for: p53 attenuates acetaminophen-induced hepatotoxicity by regulating drug-metabolizing enzymes and transporter expression
Source: Cell Death Dis. 2018 May 10;9(5):536. doi: 10.1038/s41419-018-0507-z (PMC5945795; doi:10.1038/s41419-018-0507-z)
Supplement: Supplementary file 4 — Sup Fig legend [file 41419_2018_507_MOESM4_ESM.docx]

**Supplementary Figure 1. P53 regulates *Nrf2* expression.** Since mice with a 24-hour pre-treatment of Dox were collected at 2, 6 and 24 hours after APAP exposure, we parallelly measured the expression of Nrf2 at 26, 30 and 48 hours after Dox treatment alone. (A-B) mRNA expression of Nrf2 were measured after Dox treatment alone or in p53^+/+^ and p53^-/-^ mice. Data are the mean ± S.E.M; n=5. ^**^P<0.01 versus control group.

**Supplementary Figure 2. Expression of *Nrf2* after Nrf2 siRNA transfection.** Nrf2 mRNA level was measured at 48 and 72 h after Nrf2 siRNA transfection. Data are the mean ± S.E.M; n=5. ^**^P<0.01, ^***^P<0.001 versus control group.

**Supplementary Figure 3. P53 regulates core cell cycle protein CDK4 and cyclin D1.** CDK4 and cyclin D1 protein levels were measure in p53^+/+^ and p53^-/-^ mice. Data are the mean ± S.E.M; n=5. ^**^P<0.01 versus control group.
