# Supplementary material for: p53 attenuates acetaminophen-induced hepatotoxicity by regulating drug-metabolizing enzymes and transporter expression
Source: Cell Death Dis. 2018 May 10;9(5):536. doi: 10.1038/s41419-018-0507-z (PMC5945795; doi:10.1038/s41419-018-0507-z)
Supplement: Supplementary file 5 — Sup Table 1 [file 41419_2018_507_MOESM5_ESM.pdf]

Supplemental Table 1. Primer sequence

Gene	Species	primer sequence (5′→3′)
Gapdh	Mouse	Forward: AGGTCGGTGTGAACGGATTTG
		Reverse: GGGGTCGTTGATGGCAACA
Cyp1a2	Mouse	Forward:AGTACATCTCCTTAGCCCCAG
		Reverse:GGGTCCGGGTGGATTCTTC
Cyp2e1	Mouse	Forward: CGTTGCCTTGCTTGTCTGGA
		Reverse: AAGAAAGGAATTGGGAAAGGTCC
Cyp3a11	Mouse	Forward:GGATGAGATCGATGAGGCTCTG
		Reverse: CAGGTATTCCATCTCCATCACAGT
Ugt1a1	Mouse	Forward: GCTTCTTCCGTACCTTCTGTTG
		Reverse:GCTGCTGAATAACTCCAAGCAT
Ugt1a6	Mouse	Forward: GTTTCTCTTCCTAGTGCTTTGGG
		Reverse:CCTCGTTCACTGAGATGTTCTCA
Ugt1a9	Mouse	Forward: TGTGTGGATTAATTGTCGCCA
		Reverse: CAAAGATCACTGATGGGAGCG
Sult1a1	Mouse	Forward:TGTCCTATGGGTCGTGGTACC
		Reverse:GTCTCAGCTCCCACCACTCC
Mrp2	Mouse	Forward:GTGTGGATTCCCTTGGGCTTT
		Reverse:CACAACGAACACCTGCTTGG
Mrp3	Mouse	Forward:CTGGGTCCCCTGCATCTAC
		Reverse:GCCGTCTTGAGCCTGGATAAC
Mrp4	Mouse	Forward:CATCGCGGTAACCGTCCTC
		Reverse:CCGCAGTTTTACTCCGCAG
Gstπ	Mouse	Forward:ATGCCACCATACACCATTGTC
		Reverse:GGGAGCTGCCCATACAGAC
Gstα	Mouse	Forward:AAGCCCGTGCTTCACTACTTC
		Reverse:GGGCACTTGGTCAAACATCAAA

Gstμ	Mouse	Forward:ATACTGGGATACTGGAACGTCC
		Reverse:AGTCAGGGTTGTAACAGAGCAT
Nqo1	Mouse	Forward:AGGATGGGAGGTACTCGAATC
		Reverse:AGGCGTCCTTCCTTATATGCTA
